# COX2 is involved in hypoxia-induced TNF-α expression in osteoblast

**DOI:** 10.1038/srep10020

**Published:** 2015-06-12

**Authors:** Yonggang Xing, Renxian Wang, Dafu Chen, Jianping Mao, Rui Shi, Zhihong Wu, Jun Kang, Wei Tian, Chi Zhang

**Affiliations:** 1 Department of Spine, Beijing JiShuiTan Hospital, Beijing, 100035, China; 2 Beijing Laboratory of Biomedical Materials, Laboratory of Bone Tissue Engineering, Beijing Research Institute of Traumatology and Orthopaedics, Beijing JiShuiTan Hospital, Beijing 100035, China; 3 Department of Orthopedic Surgery, Peking Union Medical College Hospital, Peking Union Medical College and Chinese Academy of Medical Sciences, Beijing, 100730, China; 4 Department of Periodontology, School and Hospital of Stomatology, Peking University, Beijing, 100081, China; 5 Bone Research Laboratory, University of Texas Southwestern Medical Center, Dallas, Texas 75390, USA

## Abstract

Bone regeneration involves a series of events in a coordinated manner, including recruitment of mesenchymal stem cells, induction of immune response, inflammatory activity and vascular ingrowth. The microenvironment of bone regeneration is hypoxic. Low oxygen tension (hypoxia) promotes the upregulation of several signaling molecules. The primary mediating factor is the hypoxia-inducible factor-1 (HIF-1). Hypoxia stimulates the expression of a variety of cytokines from inflammatory cells, fibroblasts, endothelial cells, and osteoblasts. TNF-α is a key proinflammatory cytokine. The molecular events involved in osteoblast dysfunction under hypoxia are not fully understood. This study determined the effects of hypoxia on TNF-α in osteoblasts, and molecular mechanisms were explored. We observed that hypoxia induced TNF-α expression in a time-dependent manner in osteoblasts. Experiments using a potent HIF-1α activator DFO demonstrated that hypoxia-induced TNF-α was mediated by HIF-1-α. In addition, this study showed that hypoxia activated cyclooxygenase-2 (COX2) expression along with TNF-α. Inhibition experiments using COX2 inhibitor N398 indicated that COX2 was involved in hypoxia-mediated TNF-α expression, and this observation was further confirmed by Small interfering RNA against COX2. On the other hand, TNF-α didn’t lead to the activation of COX2 expression. We conclude that COX2 is involved in hypoxia-induced TNF-α expression in osteoblast.

Bone formation is a highly regulated process that takes place during embryonic development, growth, remodeling and fracture repair^1^. Endochondral ossification and intramembranous ossification are two distinct processes of bone formation. Most bones form by endochondral ossification, which requires a cartilage intermediate. Fewer bones, such as craniofacial bones, form directly from mesenchymal condensations without cartilage template, which is called intramembranous ossification. Osteoblasts play a critical role in bone formation, and are responsible for the regulation of extracellular matrix mineralization and the control of bone remodeling. Osteoblast differentiation from mesenchymal stem cells is controlled by several important transcription factors and signaling proteins, including Indian Hedgehog, Runx2, Osterix (Osx), and Wnt pathway^2^.

Fracture healing is a complex and sequential set of events to restore bone fracture to pre-fracture condition. This is a recapitulation of molecular mechanism that controls bone formation during development and makes facture healing unique as a regenerative process than repair. Bone regeneration and fracture healing depend on different parameters present locally and systemically including growth factors, hormones, pH, oxygen tensions, immune response, etc. As in many repairs or regenerative process in the body, facture repair begins with the induction of an immune response. A hematoma is formed resulting in an inflammatory process. This is brought about by cytokines. Cytokines, such as interleukins and tumor necrosis-factor-alpha (TNF-α) secreted by inflammatory cells, have a chemotactic effect on other inflammatory cells and on the recruitment of MSCs, usually in the first three days after the facture^3^.

Angiogenesis and osteogenesis are coupled each other^4^. Blood vessels provide oxygen and nutrient for bone growth. Mesenchymal origin cells, like osteoblasts, respond to oxygen and nutrient supply level in bone. Replacing the avascular cartilage template with highly vascularized bone is the critical step of endochondral ossification. During endochondral bone formation, chondrocytes model the growth plate and become hypertrophic and hypoxic. Blood vessel invasion from the metaphyseal region into the avascular cartilage coincides with bone formation on the cartilaginous template.

Low oxygen tension or hypoxia is a pathophysiological component of many human diseases such as cancer, heart attack and stroke. Recent evidence suggests that hypoxia also plays an important role in skeletal development and cell differentiation^5^,^6^. The key mediator of the adaptive response of cells to hypoxia is the transcription factor, hypoxia-inducible factor-1 (HIF-1). HIF-1 is a heterodimer that includes HIF-1α, the oxygen sensitive subunit, and the constitutively expressed HIF-1β. Under normoxic conditions, HIF-1α is hydroxylated by prolyl hydroxylases that act as oxygen sensors. Hydroxylation of specific proline residues on HIF-1α is followed by proteasomal degradation. Under hypoxic conditions, HIF-1α is stabilized, translocated to the nucleus, and forms a dimer with HIF-1β. HIF-1 controls target gene transcription by binding to the hypoxia-responsive elements in the proximal promoter region of the oxygen responsive genes. HIF-1α is a conserved transcription factor which can activate many angiogenic genes, including VEGF. For endochondral ossification, HIF-1α upregulates VEGF, and causes enhanced bone modeling^7^. The loss of HIF-1α makes bone narrow and less vascularized. Nevertheless, VEGF was still expressed in HIF-1α null mice, indicating that besides HIF-1α, other factors are also involved in VEGF regulation during embryonic development^8^. Without angiogenesis, osteogenesis would not occur.

The molecular events involved in osteoblast dysfunction under hypoxia are not fully understood. Of particular importance in the genesis of inflammatory events are the immunomodulators referred to as cytokines^9^. Although these factors play varying roles in driving inflammatory responses, TNF-α has been demonstrated to function as a central mediator. The enzyme cyclooxygenase-2 (COX2) is responsible for the first committed step in the synthesis of several important mediators involved in both initiation and resolution of inflammation. COX2 is expressed on a wide array of stimuli, A study using adenoviral overexpression of COX2 with a mutation in the peroxidase site of COX2 led to similar susceptibility to hypoxia compared with those cells overexpressing normal COX2^10^. Current study was to determine the effects of hypoxia on TNF-α in osteoblasts under normoxic and hypoxic conditions, and molecular mechanisms were explored. We observed that COX2 is involved in hypoxia-induced TNF-α expression in osteoblast.

## Results

### Hypoxia leads to upregulation of TNF-α gene expression

**Figure 1:**
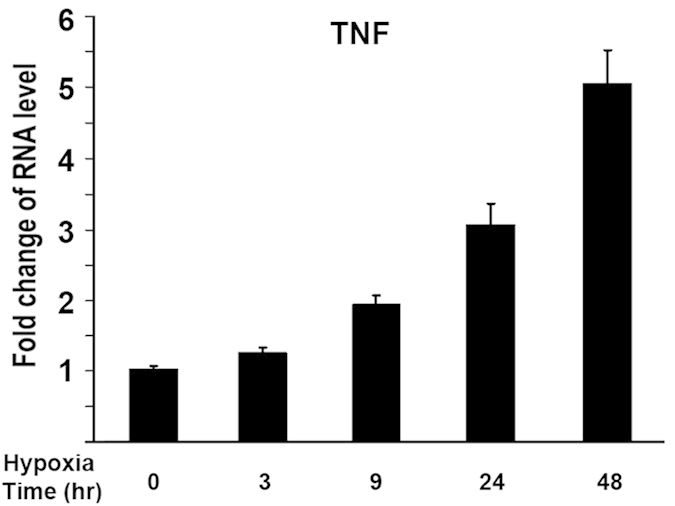
**Hypoxia leads to upregulation of TNF-α gene expression in osteoblasts.** MC3T3 osteoblasts were cultured at different time points under hypoxia (1%O_2_). RNA was isolated for analysis. RNA levels were measured by quantitative real-time RT-PCR. RNA level was normalized to heat shock protein 90 (HSP90). Level of RNA from control group was normalized to a value of 1. Values were presented as the mean ±S.D. Fold change in RNA levels compared with control group was indicated.

To explore the effect of hypoxia on expression of TNF-α in osteoblasts, we used quantitative real-time RT-PCR to examine the changes of gene expressions under hypoxia. MC3T3 osteoblastic cells were cultured and maintained in normoxic (20%O_2_) or hypoxia (1%O_2_) condition under a humidified hypoxia incubator. Total RNA was purified 48 hr following culture in the presence or absence of hypoxia. As shown in Fig. 1, TNF-α RNA expression was enhanced by 1.9 fold at 9 hr under hypoxia compared with normoxia, and enhanced by 5 fold at 48 hr after hypoxia. This demonstrated that hypoxia activates TNF-α gene expression.

### HIF-1α mediated hypoxia-induced activation of TNF-α expression

**Figure 2:**
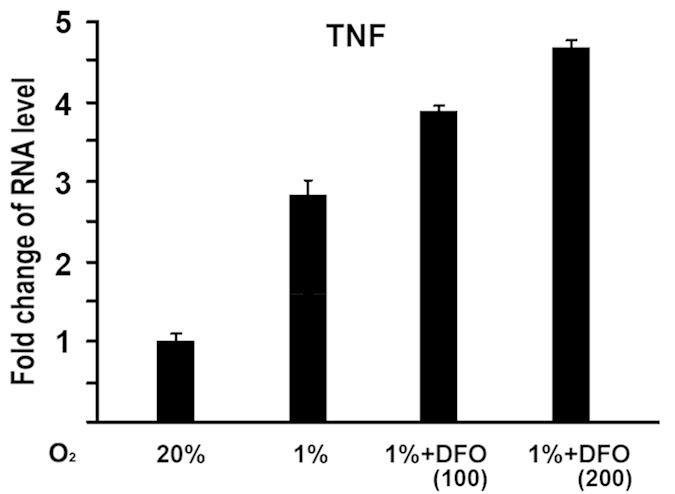
**Effect of desferrioxamine (DFO) on hypoxia-induced TNF-α expression in osteoblasts.** MC3T3 osteoblasts were cultured for 24 hr under hypoxia (1%O_2_), and treated with DFO as indicated. + :100 uM; +  + :200 uM. RNA expression level of TNF-α was determined by quantitative real-time RT-PCR. The RNA level from normoxic condition (20%O_2_) group was normalized to a value of 1. Values were presented as the mean ±S.D. Fold change in RNA levels compared with control group was indicated.

We then asked if the effect of hypoxia on TNF-α expression is related to HIF-1α. To address this question, we used DFO in this assay, a potent HIF-1α activator. TNF-α expression was then examined at 24 hr under hypoxia. As shown in Fig. 2, TNF-α expression was enhanced by 2.9 fold at 24 hr under hypoxia compared with normoxia, and additions of DFO further increased TNF-α expression under hypoxia in a dose-dependent manner. These data provided evidence that HIF-1α mediated hypoxia-induced activation of TNF-α expression.

### COX2 is involved in hypoxia-induced TNF-α activation

**Figure 3:**
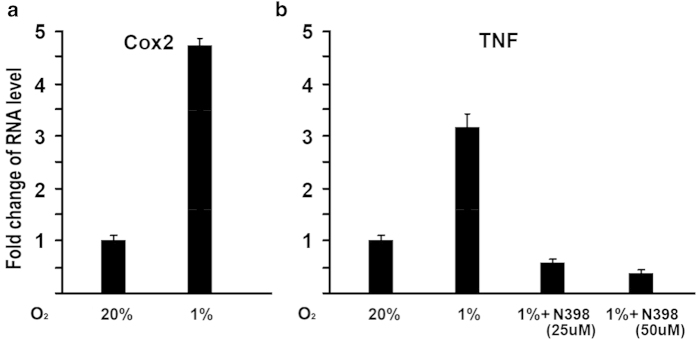
**Effect of COX2 selective inhibitor N398 on hypoxia-induced TNF-α expression in osteoblasts.** (**A**) Increase of COX2 expression in RNA level under hypoxia. MC3T3 osteoblasts were cultured for 24 hr under hypoxia (1%O_2_). RNA was isolated and quantitated by real-time RT-PCR. The RNA level from normoxic condition (20%O_2_) group was normalized to a value of 1. Values were presented as the mean ±S.D. Fold change in RNA levels compared with control group was indicated. (**B**) COX2 is involved in hypoxia-induced TNF-α activation. MC3T3 osteoblasts were cultured for 24 hr under hypoxia (1%O_2_), and treated with COX2 selective inhibitor N398 as indicated. RNA was isolated and quantitated by real-time RT-PCR. The RNA level from normoxic condition (20%O_2_) group was normalized to a value of 1. Values were presented as the mean ±S.D.

We asked whether COX2 is involved in hypoxia-induced TNF-α expression. We investigated the effects of a specific COX2 inhibitor, NS-398, on TNF-α expression under hypoxia. As shown in Fig. 3A, COX2 expression was enhanced by 4.7 fold at 24 hr under hypoxia compared with normoxia. At this condition, TNF-α expression was enhanced by 3.0 fold as shown in Fig. 3B. Interestingly, additions of specific COX2 inhibitor NS-398 dramatically inhibit TNF-α expression induced by hypoxia. These data provided evidence that COX2 is involved in hypoxia-induced activation of TNF-α expression.

### Inhibition of COX2 by siRNA leads to repression of TNF-α expression

**Figure 4:**
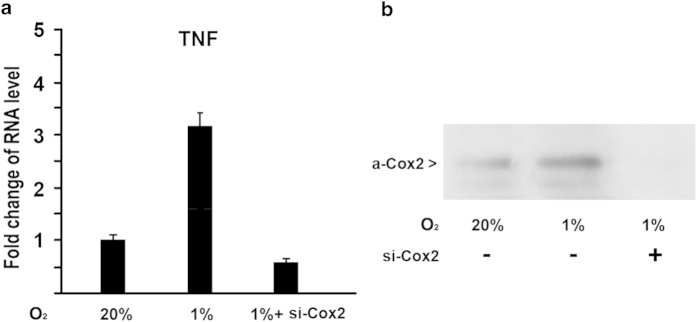
**Inhibition of COX2 by siRNA results in downregulation of TNF-α expression under hypoxia in osteoblasts.** (**A**) Effect of siRNA against COX2 on TNF-α expression under hypoxia. MC3T3 osteoblasts were transfected with siRNA against COX2. RNA was isolated 24 hr post-transfection and quantitated by real-time RT-PCR for TNF-α. The RNA level from the control group was normalized to a value of 1. Values were presented as the mean ±S.D. si-COX2: si-RNA against COX2. (**B**) Western blotting analysis of COX2 expression in protein level in osteoblasts under hypoxia. MC3T3 osteoblasts were transfected with siRNA against COX2. Protein was isolated by acetone precipitation from the cell lysates. The blot was subsequently incubated with an anti-COX2 rabbit polyclonal antibody (1:500, Santa Cruz) followed by a secondary antibody (peroxidase-conjugated anti-rabbit IgG 1:5000, Sigma). The ECL kit (Amersham Life Sciences) was used for western blot detection

To further examine the effect of COX2 on TNF-α expression induced by hypoxia, we used siRNA to knockdown COX2 expression in MC3T3 cells. MC3T3 cells were transfected by siRNA against COX2 and cultured in hypoxia (1%O_2_) condition under a humidified hypoxia incubator. Real-time RT-PCR was performed to analyze gene expression levels. As shown in Fig. 4A, TNF-α expression was induced by hypoxia at 24 hr by 3.2 fold. When siRNA targeted against COX2 was used, TNF-α levels were significantly reduced under hypoxia. Inhibition of COX2 by siRNA was confirmed by Western Blotting as shown in Fig. 4B. These loss-of-function experiments provided evidence that inhibition of COX2 leads to repression of TNF-α gene expression.

### TNF-α didn’t activate COX2 expression in osteoblasts

**Figure 5:**
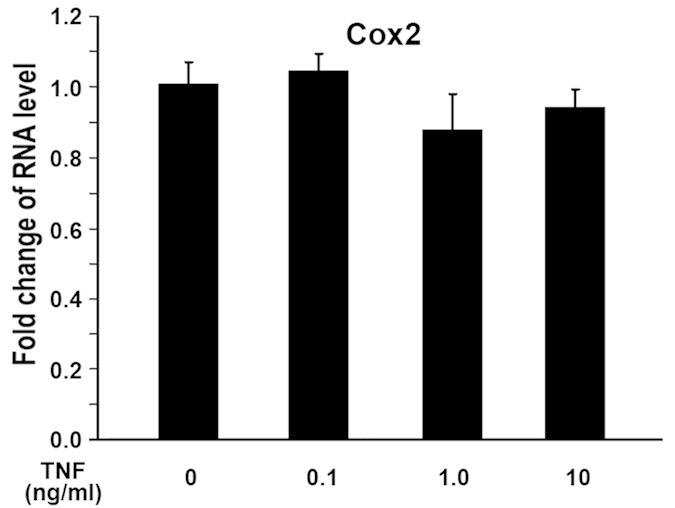
**TNF-α does not impair COX2 gene expression in MC3T3 osteoblasts.** MC3T3 osteoblasts were treated with increasing amount of TNF-α as indicated. RNA was isolated 24 hr later. RNA expression levels were determined by quantitative real-time RT-PCR. The RNA level from the control group was normalized to a value of 1. Values were presented as the mean ±S.D.

Next, we asked whether COX2 is a downstream target of TNF-α. To address this question, MC3T3 cells were stimulated by TNF-α. Cells were harvested 24 hr following the addition of TNF-α. RNA was isolated from control group and TNF-α group followed by quantitative real-time RT-PCR analysis. As shown in Fig. 5, addition of increasing amount of TNF-α didn’t activate COX2 expression. This indicates that COX2 is not a downstream target of TNF-α in osteoblasts.

## Discussion

Vascular disruption during bone fracture creates a hypoxic zone where the oxygen tension at the center of the wound is extremely low^11^. For the fracture to heal there needs to be significant cell signaling pathway to regulate and coordinate the fracture repair process. HIF pathway has been shown to be the central regulator of adaptive responses to the low oxygen availability and to be activated during fracture repair. It is suggested that hypoxia can affect osseous healing by altering the expression of cytokines, bone specific extracellular matrix molecules and their regulators. In this study, we performed a series of experiments to study the effect of hypoxia/HIF-1α on TNF-α activation in osteoblasts. The findings presented here indicate that hypoxia/HIF-1α activates TNF-α expression, and that one mechanism of hypoxia/HIF-1α effect on TNF-α expression is mediated through controlling COX2.

First, we showed that hypoxia activated TNF-α expression in osteoblasts. This was supported by in vitro assay of quantitative real-time RT-PCR. MC3T3 osteoblastic cells were cultured and total RNA was isolated for measurement of gene expressions under hypoxia. TNF-α RNA expression was enhanced under hypoxia compared with normoxia (Fig. 1). The upregulation in TNF-α RNA level under hypoxia suggests that hypoxia activates TNF-α gene expression in a time-dependent manner. HIF-1α is the key mediator of the adaptive response of cells to hypoxia. Under normoxic conditions, HIF-1α is hydroxylated by prolyl hydroxylases that act as oxygen sensors. Hydroxylation of specific proline residues on HIF-1α leads to proteasomal degradation. Under hypoxic conditions, HIF-1α is stabilized, translocated to the nucleus, and forms a heterodimer with HIF-1β to regulate target genes. These target genes are involved in a variety of cellular processes including angiogenesis, energy metabolism, cell proliferation and survival, vasomotor control, and matrix metabolism^12^. To address whether HIF-1α is involved in hypoxia-mediated activation of TNF-α expression, DFO, a potent HIF-1α activator, was used to enhance the expression of HIF-1α in this study. Figure 2 demonstrated that addition of DFO led to a further increase of TNF-α activation by Hypoxia. These experiments indicate that HIF-1α participates in hypoxia-induced activation of TNF-α in osteoblast.

Osteogenesis is the process of depositing new bone by osteoblasts. The essential step of endochondral ossification is to replace the avascular cartilage template with highly vascularized bone. Blood vessels transport mesenchymal cells to the mineralization front, where those cells differentiate to osteoblasts. VEGF is important for initial angiogenesis into the primary ossification center and keeping the blood vessel growth in developing bones^13^,^14^. VEGF is involved in blood vessel invasion of hypertrophic cartilage, and thus affects bone formation. VEGF was found to have a direct autocrine role in osteoblast differentiation^15^. VEGF can also induce angiogenesis and hence enhance oxygen and nutrients supply for osteogenesis^16^. Molecular mechanisms responsible for coupling angiogenesis and osteogenesis remain poorly understood. As one of the driving forces, hypoxia has been reported to couple angiogenesis to osteogenesis. It has been shown that constitutive activation of the HIF-1α pathway in mice promotes bone modeling and acquisition in long bones, and on the other hand, loss of HIF-1α in osteoblasts results in narrow, less vascularized bones. These results suggest that HIF-1α is critical for coupling angiogenesis to osteogenesis. Osteoblasts stay on the nascent bone surface and sense reduced oxygen or nutrient levels, and HIF-1α is an important mediator in this process.

The current study addresses possible mechanisms for hypoxia/HIF-1α to activate TNF-α expression in osteoblasts. The data indicates that hypoxia-mediated COX2 activation is one of possible mechanisms for hypoxia to activate TNF-α. This is supported by several evidences: 1) quantitative RT-PCR results showed that COX2 gene was upregulated along with TNF-α under hypoxia; 2) the treatment of COX2 inhibitor N398 abolished the activation of TNF-α expression induced by hypoxia; 3) the inhibition of COX2 by siRNA in osteoblasts led to the expression decrease of TNF-α gene under hypoxia. Therefore, the loss-of-function experiments demonstrated a role for COX2 in hypoxia-induced TNF-α gene expression. However, our study cannot rule out other possible mechanisms of the effect of hypoxia/HIF-1α on TNF-α expression. The preponderance of evidence suggests that hypoxia results in up-regulation of several molecular pathways involved in the fracture repair cascade. The influence of hypoxia/HIF-1α on the bone regenerative process will continue to be explored.

In summary, hypoxia/HIF-1α activates the expression of TNF-α through controlling COX2 expression. While further studies need to address the role of COX2 regulation by hypoxia/HIF-1α, these early stage studies revealed one mechanism through which hypoxia activates TNF-α expression in osteoblasts. Elucidation of HIF-1α activation of TNF-α will help to better understand the molecular mechanism of HIF-1α effect on osteoblasts.

## Methods

### Cell cultures and Hypoxia experiment

MC3T3 osteoblastic cells (ATCC) were cultured in Alpha Minimum Essential Medium with ribonucleosides, deoxyribonucleosides, 2mM L-glutamine and 1mM sodium pyruvate (GIBCO) and supplemented with 10% FBS and penicillin plus streptomycin as previously described^17^. Cells were cultured in 95% air/5% CO_2_ humidified incubator. Cells were trypsinized and plated before transfection. In hypoxia experiments, MC3T3 cells were maintained in Alpha Minimum Essential Medium, and cultured in normoxic (20%O_2_) or hypoxia (1%O_2_) condition incubator with 5%CO_2_ and the balanced N_2_ before harvest conclusion^18^. All endpoints measured in hypoxia cells were compared with those in cells kept under normoxic condition. Desferrioxamine (DFO) was purchased from Sigma (D9533-1G). N398 was purchased from Sigma.

### RNA isolation and Quantitative Real-time Reverse Transcription-PCR

Total RNA was isolated from MC3T3 osteoblasts with TRIzol reagent (Invitrogen) followed by RNeasy mini kit (Qiagen) as previously described^19^. TaqMan One-Step RT-PCR Master Mix reagent (Applied Biosystems) was used for quantitative RT-PCR. Reaction volume is 50 ul per well on 96-well plates. Analysis was performed with ABI PRISM 7500 sequence detection system (Applied Biosystems). Primers were ordered from Applied Biosystems. Transcript levels were normalized to heat shock protein 90 (HSP90) levels. All reactions were done in duplicate and all experiments were repeated at least three times. The relative mRNA expression levels were calculated according to the comparative C_T_ (∆∆C_T_) method as described by the manufacturer. Target quantity is normalized to endogenous control and relative to a calibrator, and is calculated using formula: Target amount = 2^−∆∆C^_T_.

### Protein purification and Western blot

Protein was isolated by acetone precipitation from the cell lysates as previously described^20^. The protein pellet was dissolved in 1% SDS buffer, warmed for 15 min at 55 °C, and centrifuged for 5 min at 14000 rpm. Protein concentrations in the supernatant were determined using a BCA Protein Assay Kit (Pierce). Proteins were separated on 10% SDS-PAGE gels and transferred to a PVDF membrane followed by Western blot analysis. Briefly, 3% milk in TBS containing 0.1% Tween-20 was used to block non-specific binding. The blot was subsequently incubated with an anti-COX2 rabbit polyclonal antibody (1:500, Santa Cruz) followed by a secondary antibody (peroxidase-conjugated anti-rabbit IgG 1:5000, Sigma). After antibody incubation, blots were extensively washed in TBS containing 0.1% Tween-20. For detection, the ECL kit (Amersham Life Sciences) was used according to the directions of the manufacturer.

### siRNA interference

MC3T3 cells were transfected by siRNA with Lipofectamine 2000 as previously described^21^. siRNA oligos against mouse COX2 were purchased from Thermo Scientific Dharmacon, and siGENOME Lamin A/C Control siRNA was used as a non-specific control. Cells were cultured in 6-well plates. One day before transfection, cells were plated in 1 ml of growth medium without antibiotics. Cells were 30-50% confluent at the time of transfection. For each sample, siRNA:Lipofectamine. 2000 transfection complex was prepared as follows: Dilute 2 μl of 50 μM siRNA in 50 μl of Opti-MEM I Reduced Serum Medium without serum; Mix Lipofectamine. 2000 gently, then dilute 3 μl in 50 μl of Opti-MEM I Medium; Combine the diluted siRNA with the diluted Lipofectamine. 2000; and then add 100 μl of siRNA:Lipofectamine. 2000 complex to each well. After 4 hr incubation, the growth medium was replaced. Cells were cultured at 37 °C in a CO_2_ incubator for 24 hr before harvest.

### Statistical Analysis

All experiments were repeated a minimum of 3 times. Data was reported as the mean ± standard deviation (S.D.). Comparisons were made between groups by Student’s t test with p < 0.05 being considered as statistically significant.

## Additional Information

**How to cite this article**: Xing, Y. *et al*. Cox2 is involved in hypoxia-induced TNF-α expression in osteoblast. *Sci. Rep.* **5**, 10020; doi: 10.1038/srep10020 (2015).
